# Antimicrobial Resistance Trends of *Escherichia coli* Isolates: A Three-Year Prospective Study of Poultry Production in Spain

**DOI:** 10.3390/antibiotics11081064

**Published:** 2022-08-05

**Authors:** Sandra Sevilla-Navarro, Pablo Catalá-Gregori, Jan Torres-Boncompte, Maria Teresa Orenga, Josep Garcia-Llorens, Verónica Cortés

**Affiliations:** 1 Centro de Calidad Avícola y Alimentación Animal de la Comunidad Valenciana (CECAV), 12539 Castellón, Spain; 2 Departamento de Producción y Sanidad Animal, Salud Pública Veterinaria y Ciencia y Tecnología de los Alimentos, Instituto de Ciencias Biomédicas, Facultad de Veterinaria, Universidad Cardenal Herrera-CEU, CEU Universities, 46113 Moncada, Spain; 3 Laboratorios ALS-TACSA, 46470 Valencia, Spain; 4 Facultad de Medicina, Departamento de Medicina Preventiva y Salud Pública, Universitat de Valencia, 46010 Valencia, Spain

**Keywords:** *Escherichia coli*, antimicrobial trends, multidrug resistance, monitoring

## Abstract

Antimicrobial resistance (AMR) poses a major threat to health worldwide. Poultry products are one of the main threats, due to the transmission of antimicrobial resistance genes throughout the food chain. *Escherichia coli* is the main cause of mortality in the poultry industry, mainly mitigated with antibiotics, but due to the high genetic strain variability, recurrent outbreaks of multidrug resistant *E. coli* take place. The major challenge to tackling AMR is understanding the burden of resistance. For this reason, one of the main strategies is monitoring AMR by phenotypic characterisation. Our study aimed to monitor the resistance of *E. coli* strains isolated from the poultry sector over a period of three years (2019–2021) to provide information on the resistance magnitude and trends. Promising results have been found concerning the low frequency of resistance to cephalosporins, polymyxin, and fluoroquinolones. However, levels of resistance found to antimicrobials such as erythromycin (100%), tylosin (98%), or penicillin (97%) suggest the need to continue working on the limitation of use of antimicrobials in poultry to achieve the demise of MDR.

## 1. Introduction

Antimicrobial resistance (AMR) has emerged as one of the major threats to public and veterinary health worldwide [1]. The latest review on antimicrobial resistance estimated that AMR could kill 10 million people per year by 2050 [1,2]. Livestock production is an ongoing concern regarding the spread of resistant bacteria from animals to humans through contaminated meat products [2,3]. In fact, according to the European Food Safety Authority (EFSA), animal food products such as unpasteurized (raw) milk and undercooked meat or eggs are some of the main sources of contamination of zoonotic agents in humans [4]. This might increase the risk of exposure to AMR bacteria from farm to fork [3,5]. For this reason, one of the main concerns of the poultry sector is to control and prevent the transmission of such microorganisms through the food chain [6] by continued monitoring and surveillance. 

*Escherichia coli* (*E. coli*) is one of the most widely distributed microorganisms of the intestinal tract of warm-blooded animals, such as mammals and chickens [7,8]. Main *E. coli* isolates are not pathogenic but have enhanced fitness of pathogenicity, effective transmission and colonisation abilities, global distribution due to efficient dissemination, and resistance to various antimicrobials [9]. In addition, a subgroup could cause extraintestinal infections due to Extraintestinal Pathogenic *E. coli* (ExPEC) and, more particularly, a subset of the ExPEC group is Avian Pathogenic *E. coli* (APEC), responsible for colibacillosis in avian species, which is one of the main causes of mortality in poultry [10,11]. To comply with animal welfare standards and prevent animals suffering distress, *E. coli* is mainly mitigated with antibiotics. Nevertheless, due to the high genetic strain variability and the presence of resistance, recurrent outbreaks of multidrug resistance (MDR) *E. coli* take place [12]. 

In 2017, the World Health Organisation (WHO) published the list of 12 priority antibiotic-resistant pathogens, among which *E. coli* was included [13]. Poultry farming is an important reservoir of virulent pathogenic *E. coli* due to the presence of antibiotic resistance genes (ARGs) [14]. High levels of resistance have been detected to fluoroquinolones and extended-spectrum cephalosporins [15], compounds that have been classified by the WHO as antibiotics of “critical importance and highest priority” for human medicine due to the limited availability of alternatives for the treatment of bacterial infections. This situation has led to significant restrictions on the use of antibiotics. Thus, in 2011, the European Commission called upon all European Member States (MS) to develop an action plan on antimicrobial resistance for a joint approach to this problem. In this regard, since 2014, in Spain, the National Antibiotic Resistance Plan (PRAN) has been implemented by the poultry sector, which is committed to reducing antibiotic use at the field level [16]. 

The major challenge in tackling AMR is understanding the burden of resistance [1]. For this reason, one of the main strategies followed by the PRAN is phenotypic characterisation when antibiotic use is necessary, with the aim of reducing and rationalising the use of antibiotic therapy. 

In this context, due to the importance of this microorganism both in animal and public health, the aim of our study was to monitor the resistance of *E. coli* strains isolated from the poultry sector over a three-year period (2019–2021) to provide information on the resistance magnitude and trends.

## 2. Results

**Figure 1:**
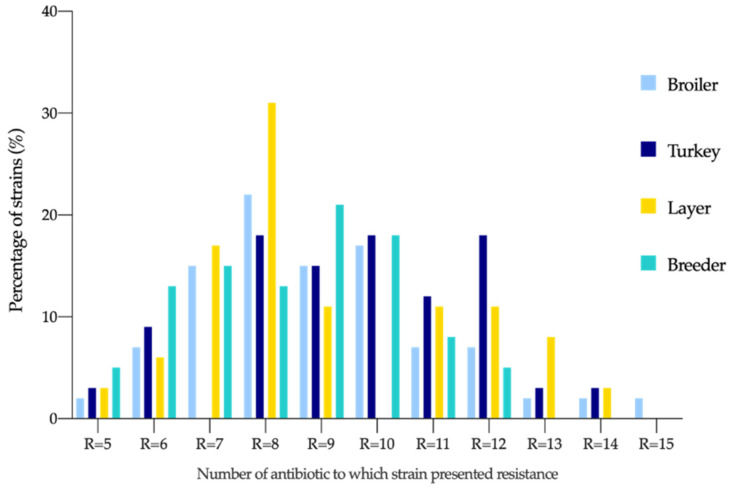
Resistance distribution according to different poultry production types. R = Number of antimicrobial resistances.

All isolates analyzed in this study were MDR (274; 100%), and 5% (13/274) were extremely drug resistant (XDR), from which 54% of the isolates (7/13) were from broilers, 30% from turkeys (4/13), and 8% from layers (1/13) and breeders (1/13). The MDR rate in all production orientations exhibited resistance to more than five different antibiotics, with 15 as the maximum number of resistances found, in broilers, followed by 14 in turkeys and layers and 12 in breeders (Figure 1).

Using a generalized linear model, statistically significant differences were observed between the different antimicrobials, with the highest resistance values being observed against erythromicin (ERY) (274/274; 100%), tylosin (TLS) (269/274; 98%), penicillin (PEN) (267/274; 97%), oxacycline (OXA) (64/274; 96%), tiamulin (TIA) (250/274; 91%), lincomysin-spectinomycin (LIS) (234/274; 85%), tilmicosin (TILM) (225/274; 82%), and trimethoprim-sulfamethoxazole (T/S) (181/274; 66%) (*p*-value = 0.000). Concerning percentage of AMR rates within each year, a statistically significant decrease in resistance was observed in LIS, TILM, amoxicillin (AMX), doxycyline (DOX), tetracycline (TET), colistin (COL) and T/S, with lincomysin (LIN) as the only antibiotic that exhibited an increase in AMR rates (*p*-value < 0.05). Levels of resistance observed for each antibiotic are presented in Table 1.

AMR rates for each livestock production and year are presented in Table 2. Broiler *E. coli* isolates presented high frequency of resistance (>80% of isolates) to ERY (100%), PEN (99%), OXA (98%) and TIA (91%) and low frequency (<10% of isolates) of resistance to ceftiofur (CET) (4%), cefpodoxime-proxetil (CPP) (5%), neomycin (NEO), and COL (7%). In turkeys, there was a high frequency of resistance to ERY (100%), TLS (100%), PEN (94%), and OXA (84%) and low frequency of resistance to CPP (3%), CET (5%), NEO (6%), ENRO, and CTX (8%) was found. In layers, there was a high frequency of resistance of the *E. coli* isolates to ERY (100%), TLS (100%), OXA (98%), TIA (95), and PEN (96%). However, a lower frequency of resistance was found to NEO (2%), CPP (8%), CTX, and CET (9%). Concerning breeders, similar rates of resistance were shown; *E. coli* isolates presented high frequency of resistance to ERY and TLS (98%), PEN and OXA (96%) and TIA (94%), and low frequency of resistance to CPP (0%), NEO (2%), ENRO (4%), and CTX (7%). 

The MDR phenotypic patterns of *E. coli* are shown in Table 3. A total of 36 different patterns replicated in two or more strains were identified. The most predominant MDR phenotypes were PEN-OXA-TLS-TILM-ERY-LIS-T/S-TIA (*n* = 26), followed by PEN-OXA-TLS-TILM-ERY-LIS-TIA (*n* = 16), and PEN-AMX-OXA-TLS-TILM-ERY-LIS-T/S-TIA (*n* = 11).

**Figure 2:**
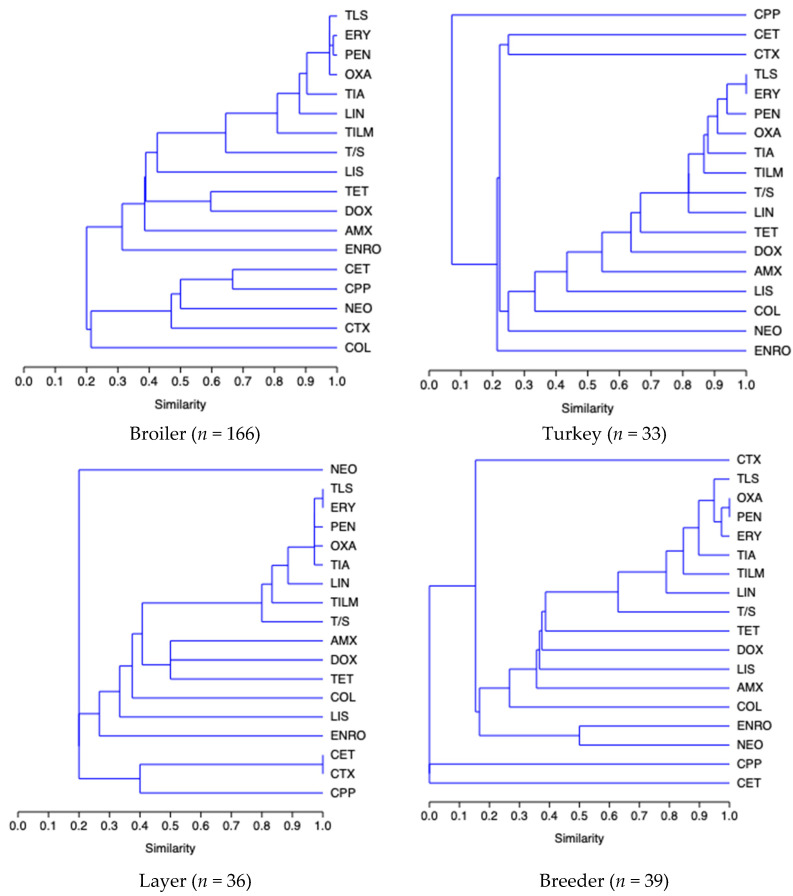
Single-linkage clustering dendrograms of resistance of *E. coli* isolates to antimicrobials by each poultry livestock production type. PEN: penicillin; AMX: amoxicillin; OXA: oxacycline; ENRO: enrofloxacinm; CET: ceftiofur; CPP: cefpodoxime-proxetil; CTX: cefotaxime; TLS: tylosin; TILM: tilmicosin; ERY: erythromycin; NEO: neomycin; COL: colistin; TET: tetracycline; DOX: doxycycline; TIA: tiamulin; LIN: lincomycin; LIS: lincomycin-spectinomycin; T/S: trimethoprim-sulfamethoxazole.

Single-linkage clustering dendrograms with Jaccard distances for *E. coli* resistance are presented in Figure 2. A relatively high proportion of the broiler *E. coli* isolates were resistant to PEN and OXA, PEN and TLS (similarity = 0.96), TLS and OXA (similarity = 0.95) and ERY and TIA, TIA and OXA (similarity = 0.90). In turkeys, high similarity was found in PEN and ERY and PEN and TLS cluster (similarity = 0.93) and TLS and OXA (similarity = 0.90). In layers, another cluster included TLS and ERY (similarity = 1), PEN and OXA and TIA (similarity = 0.94), PEN and TLS, TLS and TIA, and OXA and ERY (similarity = 0.97). Finally, in breeders, a cluster included OXA and PEN (similarity = 0.96), PEN and ERY (similarity = 0.97), PEN and TLS, TLS and ERY, (similarity = 0.92), and CET and CPP (similarity = 1). 

## 3. Discussion

The present study assessed the AMR trends of *E. coli* isolates from clinical colibacillosis over three years. Results obtained in this study highlight a high frequency of antimicrobial resistance rates to main antibiotics used in poultry production, although there was a very low frequency of resistance to antimicrobials classified as 3rd and 4th generation. The fact of finding a very low frequency of resistance to cephalosporins, polymyxin, and fluoroquinolones is encouraging from the “One Health” point of view, as the WHO categorizes these antimicrobials as the highest priority critically important antimicrobials (HPCIA) [13,15]. These results are in accordance with the last report published by the PRAN and the European Surveillance of Veterinary Antimicrobial Consumption (ESVAC), as a significant reduction in sales of veterinary medicines is shown [16,17]. 

In most developed countries, livestock uses 50–80% of antimicrobials produced [18], with TET (32%), PEN (26%), sulphonamides (12%), macrolides (7%), polymyxins (5%), and aminoglycosides (5%) as the most commonly used antibiotics. Antibiotic use increases the selection pressure of AMR bacteria [19]; in fact, in this study, 100% of the isolates were MDR and 5% XDR. Results of this work showed high resistance rates against some of the antibiotics included in macrolides (ERY 100%, TILM 82%, TLS 98%), penicillins (PEN 97% and OXA 96%), pleuromutilins (TIA, 91%), and lincosamides (LIN 85%), suggesting that these strains are an important reservoir of resistance genes [20]. Except for TILM, the resistance trend in these previous antibiotics has remained at high levels during these years or has increased. This high incidence of MDR is extremely significant, involving a serious health risk, as MDR isolates may have the chance of contaminating food products and being transferred to humans. Different studies have demonstrated the close association between animal products, such as poultry derivatives, and humans [21,22,23,24,25,26]. 

On the other hand, a downward trend in the use of AMX, DOX, TET, and COL has been observed, highlighting the reduction of COL-AMR rates. These results are in accordance with those published by the EFSA, as a decreasing trend in resistance not only to COL but also to TET has been described [15]. However, different results were obtained by Jamal et al. and Racewicz et al., who reported a high percentage of *E. coli* strains resistant to TET (97%) and (86%; particularly to DOX), respectively [18,27]. This is not surprising, as TET are commonly used to treat bacterial infections in poultry [28,29]. Concerning percentage AMR rates of COL, differences have been observed between values reported [30] and ours, obtaining resistance percentages of 42.56% and 25%, respectively. In addition, results for COL resistance in this study are higher (7%) than those published by the EFSA and ECDC for broilers in Spain (0%) and in the EU (0.7%). Lower rates of COL compared to other antimicrobials in this study should be highlighted, as this antimicrobial is used as a last resort antibiotic in humans and, since the discovery of the *mcr-1* plasmid gene in animal food, this leads to a worldwide concern over horizontal transfer of resistance genes in humans [31]. In agreement, the Spanish government implemented a specific plan for the reduction of colistin in the swine sector that was also implemented in poultry farming, achieving a significant reduction of 71% in poultry meat farming [16]. 

Nevertheless, a significant increase of AMR in LIN has been reported, which agrees with the data published by the PRAN in Spain, as a slight upturn in sales of this antimicrobial has been observed, together with the consumption of extended-spectrum PEN, TET, LIN, sulphonamides, and macrolides. LIN is one of the antimicrobials indicated for use in chickens in the treatment of respiratory disease caused by *E. coli* [32], and it is noteworthy that the increased rates observed in our study were reported in three of the poultry production livestock operations. 

Concerning AMR rates for each livestock production, no significant differences were shown between percentage rates of resistance, except in the case of turkeys, which presented the highest percentage of resistance for both DOX (26%) and COL (25%). The ESFA and European Centre for Disease Control (ECDC) reported higher rates of AMR in turkey production in different European Countries (EC) [15]. This could be due to the fact that the farming conditions of turkeys may affect their natural immunity, making them more susceptible to environmentally conditioned diseases, such as *E. coli*, requiring antimicrobial treatment, which would in turn promote AMR [33].

Our cluster analysis of the *E. coli* isolates showed that there was concurrent resistance to ERY and PEN in the broilers, turkeys, and layers. In addition, the same antimicrobial pattern concerning TLS and ERY in the layers and OXA and PEN in breeders was identified. These strong correlations in resistance underscore the need for prudent use of antimicrobials to limit the spread of MDR bacteria in poultry flocks [34,35].

Colibacillosis is the leading cause of mortality (up to 20%) in the poultry industry and its prevalence is up to 36.7% [12,36]. Antibiotics are used to treat poultry colibacillosis cases, especially in the first week of life when the immune system of the animals is not fully developed [37]. MDR *E. coli* strains are a major problem, as due to the high variability observed, *E. coli* outbreaks happen on the same farm despite the massive use of antibiotics [12]. This entails a high risk for both animal and human medicine and poses a zoonotic threat either by causing disease in the human host or by facilitating the spread of AMR genes to pathogens, such as *Salmonella* and *Campylobacter*. These pathogens are the most reported zoonotic disease in EC, with domestic poultry as the main reservoir [15,30,38].

Infections caused by multidrug-resistant (MDR) pathogens have a significant impact on healthcare systems and economic productivity [39]. Antimicrobial misuse in livestock constitutes a direct link between animals and humans, being one of the main contributors [40]. They have been used for growth promotion in livestock, more precisely in poultry, as well as prophylaxis and therapeutics, encouraging the emergence of resistant strains. Thus, in 2006, the European Union (EU) banned the use of antibiotics for growth promotion purposes in livestock [40] and began to limit and control their use in livestock. Despite their control, the rate of resistance to certain classes of antibiotics continues to rise [40] and different studies have demonstrated the close association between animal products and humans [21,22,23,24,25,26]. Therefore, for a long time, AMR has been centered on the clinical field, but currently it is widely recognized as a problem that affects human, animals, and the environment, which needs to be addressed from a One Health approach [37]. In fact, the overall analysis performed of antibiotic consumption and resistance, both in humans compared to humans, animal compared to animal and animal compared to human, shows the existence of positive correlations between antibiotic consumption and resistance rates, with a maximum for animal compared to animal and a minimum for animal compared to human. These correlations have been established as ecological, not indicating a cause-effect [16].

Thus, interventions such as monitoring campaigns could help us to analyze the evolution of AMR, to be able to implement strategies to reduce the dissemination pathway through the food chain.

## 4. Materials and Methods

### 4.1. Study Design

**Figure 3:**
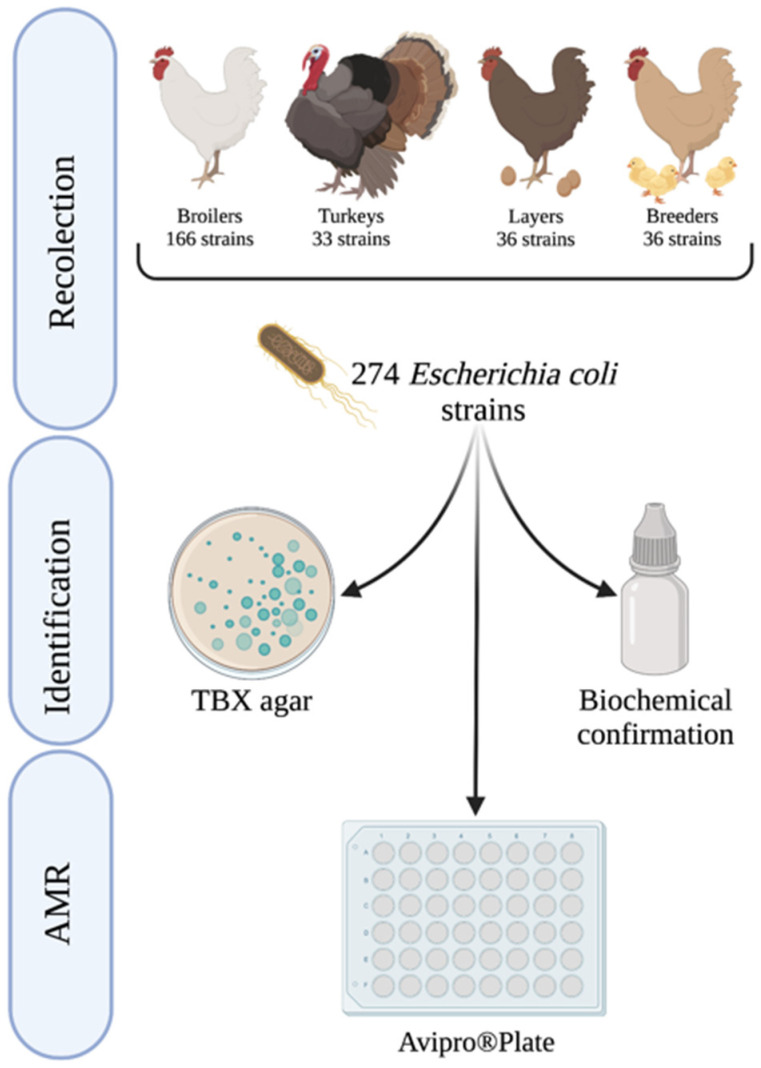
Scheme of the *E. coli* analysis performed in the study by year and livestock production. AMR: antimicrobial resistance; TBX: Tryptone Bile X-Glucuronide (Created with Biorender).

Samples were obtained from viscera swabs of animals with compatible symptoms of colibacillosis from poultry farms located in Spain between January 2019 and December 2021. A total of 274 *E. coli* strains were analyzed (92 in 2019; 93 in 2020; 89 in 2021). Strains were isolated from broilers (166 strains), turkeys (33 strains), layers (36 strains), and breeders (39 strains). Figure 3 details the samples analyzed by year and type of poultry production.

### 4.2. Microbiological Isolation of E. coli 

*E. coli* strains were obtained using cloacal swabs from animals with compatible symptoms of colibacillosis. These sample swabs were cultured directly in non-specific media: blood agar (Oxoid, Madrid, Spain) under aerobic and anaerobic conditions and in chromogenic medium Tryptone Bile x-glucuronide (TBX) Agar (VWR, Leuven, Belgium). Seeded plates of Blood Agar were incubated at 37 ± 1 °C for 24 ± 3 h and the plates of TBX were incubated at 44 °C 24 ± 3 h. After incubation, suspected colonies were seeded on Nutrient Agar (VWR, Leuven, Belgium) and incubated at 37 ± 1 °C for 24 ± 3 h. Biochemical confirmation was performed using API galleries (API-20^®^, bioMérieux, Barcelona, Spain). Isolated strains were kept frozen at −80 °C after resuspending them in sterile distilled water with 20% (*v*/*v*) bidistilled glycerol 99.5% (VWR, Leuven, Belgium).

### 4.3. Antimicrobial Susceptibility Testing and Classification

Antimicrobial susceptibility testing was performed using Avipro^®^PLATE plates (Lohmann Animal Health GmbH, Cuxhaven, Germany). These plates are a simplified version of the classic broth dilution-based susceptibility testing method. The tested antibiotics are those for specific use in poultry, including the following families: β-lactam: penicillin (PEN, 0.125–2 µg/mL), amoxicillin (AMX, 2–16 µg/mL) and oxacycline (OXA, 0.25–2 µg/mL); fluoroquinolones: enrofloxacin (ENRO, 0.25–2 µg/mL); cephalosporins: ceftiofur (CET, 2 µg/mL), cefpodoxime-proxetil (CPP, 4 µg/mL), and cefotaxime (CTX, 1 µg/mL); macrolides: tylosin (TLS, 0.5–1 µg/mL), tilmicosin (TILM, 8–16 µg/mL), erythromycin (ERY, 0.25–4 µg/mL); aminoglycosides: neomycin (NEO, 8–16 µg/mL); polymyxins: colistin (COL, 2–4 µg/mL); tetracyclines: tetracycline (TET, 2–8 µg/mL) and doxycycline (DOX, 2–4 µg/mL); pleuromutilins: tiamulin (TIA, 8–16 µg/mL); lincosamides: lincomycin (LIN, 4 µg/mL) and Lincomycin–Spectinomycin (LIS, 8–32 µg/mL); Folate inhibitors; trimethoprim-sulfamethoxazole combination (T/S, 0.5/9.5–2/38). *E. coli* isolates with a minimum inhibitory concentration lower than or equal to the susceptible breakpoint were classified as susceptible, whereas those with a minimum inhibitory concentration higher than the susceptible breakpoint were resistant. For the recovery of the frozen strains, 10 µL of frozen suspension was sown on solid nutritive agar (Biokar^®^, Allonne France). Subsequently, the strains were incubated at 37 ± 1°C for 24 ± 3 h. After the strains’ growth, the microtitre plates Avipro^®^PLATE (Lohmann Animal Health GmbH, Cuxhaven, Germany) were inoculated and interpreted following the manufacturer’s instructions. *E. coli* strain CECT 434 was used as a positive quality control. An isolate was defined as MDR if it was resistant to at least three or more antimicrobial families of antibiotics. An isolate was defined as Extremely Drug Resistant (XDR) if it was non-susceptible to at least one agent in all but two or fewer antimicrobial categories [41]. 

### 4.4. Statistical Analysis

A generalized linear model (GLM) was used to compare the AMR rates of each antibiotic within the same type of poultry production throughout the years (2019, 2020, and 2021) and to compare the global results between each poultry production type (broilers, turkeys, layers, and breeders). This model was also used to analyze the AMR rates of each antibiotic within the same year. A *p*-value of ≤0.05 was considered a statistically significant difference. To compare individual antimicrobials with respect to their similarity in the resistance status of *E. coli*, a cluster analysis, using the Jaccard binary similarity coefficient, was performed for each poultry species. Dendrograms were constructed using the single-linkage clustering method with the Jaccard distance. The Jaccard distance measures dissimilarity between antimicrobials and is obtained by subtracting the Jaccard binary similarity coefficient from one. This analysis was selected according to Varga et al. and Kaufman et al. [35,42]. Analyses were carried out using a commercially available software application (SPSS 24.0 software package; SPSS Inc., Chicago, IL, USA, 2002). 

## 5. Conclusions

Overall, levels of resistance found in this study suggest the need to continue working on the limitation of use of antimicrobials in poultry to achieve the demise of MDR. The finding of a low frequency of resistance to cephalosporins, polymyxin, and fluoroquinolones is a very promising result that shows that the poultry sector is making efforts to reduce the use of antibiotics through improved management and safety measures. However, there are still high levels of resistance to other antibiotics such as macrolides, penicillin, and lincosamides.

Thus, standardized and continuous surveillance programs, rapid diagnostics, or the promotion of antimicrobial alternatives are necessary to monitor and tackle the occurrence and persistence of AMR isolated in the poultry sector (ESVAC) [17]. For this reason, monitoring its AMR rates should be considered not only to achieve treatment success but also to track emerging resistance in livestock and possible spread to animal-derived foods.

To this end, the Spanish poultry sector voluntarily agreed to join the REDUCE national program in 2020 to develop new and more effective preventive health plans designed to reduce total antibiotic consumption by 45% in broiler production. In addition, new programs for laying hens and turkeys have been consolidated and efforts are being made to reduce the consumption of antimicrobials over a period. 

## Figures and Tables

**Table 1:** Percentage of *E. coli* AMR strains by year.

Antimicrobial Class	Antimicrobial	Year (%; CI)			$\overset{-}{x}$
		2019 *n* = 92	2020 *n* = 93	2021 *n* = 89	
β-Lactams	PEN	97 (0.91–0.99)	99 (0.94–1)	97 (0.91–0.99)	97 ^G^ (0.95–0.99)
	AMX	35 ^ab^ (0.05–0.45)	45 ^b^ (0.35–0.55)	26 ^a^ (0.18–0.36)	35 ^A^ (0.3–0.41)
	OXA	99 (0.94–1)	95 (0.88–0.98)	96 (0.89–0.98)	96 ^G^ (0.94–0.98)
Fluoroquinolones	ENRO	17 (0.11–0.26)	12 (0.06–0.20)	10 (0.05–0.18)	13 ^C^ (0.1–0.18)
Cephalosporin	CET	4 (0.02–0.10)	6 (0.03–0.13)	3 (0.01–0.09)	5 ^B^ (0.03–0.08)
	CPP	2 (0.00–0.07)	6 (0.03–0.13)	4 (0.02–0.11)	4 ^B^ (0.02–0.07)
	CTX	11 (0.06–0.19)	11 (0.06–0.18)	7 (0.03–0.14)	9 ^C^ (0.06–0.13)
Macrolides	ERY	100 (0–1)	99 (0.94–1)	100 (0–1)	100 ^C^ (0.98–1)
	TILM	97 ^b^ (0.91–0.99)	77 ^a^ (0.68–0.85)	72 ^a^ (0.62–0.80)	82 ^D^ (0.77–8.86)
	TLS	97 (0.91–0.99)	100 (0–1)	98 (0.92–1.00)	98 ^EG^ (0.96–0.99)
Tetracyclines	DOX	43 ^b^ (0.33–0.53)	18 ^a^ (0.11–0.27)	20 ^a^ (0.13–0.30)	27 ^D^ (0.22–0.33)
	TET	49 ^b^ (0.39-0.59)	34 ^a^ (0.25–0.44)	31 ^a^ (0.23–0.42)	38 ^A^ (0.33–0.44)
Aminoglycosides	NEO	9 (0.04–0.15)	6 (0.03–0.13)	1 (0.00–0.06)	5 ^B^ (0.03–0.08)
Lincosamides	LIN	66 ^a^ (0.45–0.65)	92 ^b^ (0.16–0.33)	98 ^b^ (0.23–0.42)	85 ^A^ (0.31–0.43)
	LIS	55 ^b^ (0.56–0.75)	24 ^a^ (0.86–0.97)	31 ^a^ (0.92–1.00)	37 ^B^ (0.81–0.89)
Folate inhibitors	T/S	78 ^a^ (0.69–0.86)	62 ^a^ (0.52–0.72)	57 ^b^ (0.47–0.67)	66 ^H^ (0.6–0.71)
Polymyxins	COL	17 ^b^ (0.09–0.24)	11 ^ab^ (0.06–0.18)	4 ^a^ (0.02–0.11)	11 ^C^ (0.07–0.14)
Pleuromutilins	TIA	95 (0.88–0.98)	90 (0.83–0.95)	89 (0.81–0.94)	91 ^I^ (0.87–0.94)

$\overset{-}{x}$: period mean; ^ab^: different lower case letters in superscript represent significant differences for each antimicrobial between years. ^A–E,G–I^: different capital case letters in superscript represent significant differences within antimicrobials (*p*-value ≤ 0.05). PEN: penicillin; AMX: amoxicillin; OXA: oxacycline; ENRO: enrofloxacin; CET: ceftiofur; CPP: cefpodoxime-proxetil; CTX: cefotaxime; TLS: tylosin; TILM: tilmicosin; ERY: erythromycin; NEO: neomycin; COL: colistin; TET: tetracycline; DOX: doxycycline; TIA: tiamulin; LIN: lincomycin; LIS: lincomycin-spectinomycin; T/S: trimethoprim-sulfamethoxazole.

**Table 2:** Percentage of AMR rates of *Escherichia coli* strains.

	BROILER *n* = 166				TURKEY *n* = 33				LAYER *n* = 36				BREEDER *n* = 39			
	(%)				(%)				(%)				(%)			
	2019	2020	2021	$\overset{-}{x}$	2019	2020	2021	$\overset{-}{x}$	2019	2020	2021	$\overset{-}{x}$	2019	2020	2021	$\overset{-}{x}$
PEN	100	100	97	99	83	100	100	94	100	100	88	96	95	93	100	96
AMX	38 ^b^	56 ^b^	18 ^a^	37	25 ^a^	25 ^a^	69 ^b^	40	55	41	13	36	21	21	33	25
OXA	100	98	95	98	100	75	92	89	100	94	100	98	95	93	100	96
ENRO	24	15	10	16	17	0	8	8	9	12	25	15	5	7	0	4
CET	4	6	3	4	8	0	8	5	9	18	0	9	0	0	0	0
CPP	4	7	3	5	0	0	8	3	0	12	13	8	0	0	0	0
CTX	12	11	8	10	25 ^b^	0 ^ab^	0 ^a^	8	9	18	0	9	0	7	17	8
ERY	100	100	100	100	100	100	100	100	100	100	100	100	100	93	100	98
TILM	96 ^b^	80 ^a^	69 ^a^	82	100 ^b^	63 ^a^	77 ^a^	80	100	71	88	86	95	86	100	82
TLS	96	100	97	98	100	100	100	100	100	100	100	100	95	100	100	98
DOX	44 ^b^	19 ^a^	16 ^a^	26 ^A^	67	25	46	46 ^B^	45 ^b^	12 ^a^	0 ^a^	19 ^A^	26	21	33	27 ^A^
TET	46	33	27	36	75	50	54	60	55	29	38	40	37	36	17	30
NEO	10	9	2	7	17	0	0	6	0	6	0	2	5	0	0	2
LIN	72 ^a^	91 ^b^	98 ^b^	87	50 ^a^	100 ^b^	100 ^b^	83	76	94	88	85	58 ^a^	93 ^b^	100 ^b^	84
LIS	66 ^b^	28 ^a^	31 ^a^	41	75 ^b^	25 ^a^	15 ^a^	38	36	18	38	31	26 ^ab^	14 ^a^	67 ^b^	36
T/S	78 ^b^	59 ^a^	58 ^a^	65	83	88	77	83	100 ^b^	53 ^a^	50 ^a^	68	63	71	17	50
COL	10	7	5	7 ^A^	42	25	8	25 ^B^	18	12	0	10 ^AB^	21	14	0	12 ^AB^

$\overset{-}{x}$: period mean; ^ab^: For each antibiotic, values within the same production orientation in different years with different lower-case letters in superscript are significantly different (*p* ≤ 0.05). ^A,B^: For each antibiotic, mean values within each production orientation with different capital case letters in superscript are significantly different (*p* ≤ 0.05). PEN: penicillin; AMX: amoxicillin; OXA: oxacycline; ENRO: enrofloxacin; CET: ceftiofur; CPP: cefpodoxime-proxetil; CTX: cefotaxime; TLS: tylosin; TILM: tilmicosin; ERY: erythromycin; NEO: neomycin; COL: colistin; TET: tetracycline; DOX: doxycycline; TIA: tiamulin; LIN: lincomycin; LIS: lincomycin-spectinomycin; T/S: trimethoprim-sulfamethoxazole.

**Table 3:** Phenotype resistance profile of *E. coli* isolates (*n* = 175) in poultry flocks.

Antimicrobial nº	Antimicrobial Resistance Pattern	*n*	%
15	PEN-AMX-OXA-ENRO-CET-CPP-CTX-TLS-TILM-ERY-DOX-NEI-COL-LIS-TIA	2	1.1
13	PEN-AMX-OXA-ENRO-TLS-TILM-ERY-TET-DOX-LIS-LIN-T/S -TIA	2	1.1
	PEN-AMX-OXA-TLS-TILM-ERY-TET-DOX-COL-LIS-LIN-T/S-TIA	3	1.7
12	PEN-AMX-OXA-ENRO-TLS-TILM-ERY-TET-DOX-LIS-T/S-TIA	2	1.1
	OXA-TLS-TILM-ERY-TET-DOX-NEO-COL-LIS-LIN-T/S-TIA	2	1.1
	PEN-OXA-ENRO-TLS-TILM-ERY-TET-DOX-LIS-LIN-T/S-TIA	3	1.7
	PEN-AMX-OXA-TLS-TILM-ERY-TET-DOX-LIS-LIN-T/S-TIA	7	4.0
11	PEN-AMX-OXA-TLS-TILM-ERY-TET-DOX-LIS-T/S-TIA	4	2.3
	PEN-OXA-TLS-TILM-ERY-TET-DOX-LIS-LIN-T/S-TIA	5	2.9
	PEN-AMX-OXA-TLS-TILM-ERY-DOX-LIS-LIN-T/S-TIA	2	1.1
10	PEN-AMX-OXA-TLS-TILM-ERY-TET-LIS-T/S-TIA	2	1.1
	PEN-OXA-TLS-TILM-ERY-DOX-COL-LIS-T/S-TIA	2	1.1
	PEN-AMX-OXA-TLS-TILM-ERY-TET-LIS-LIN-TIA	4	2.3
	PEN-OXA-TLS-TILM-ERY-TET-DOX-LIS-T/S-TIA	4	2.3
	PEN-AMX-OXA-TLS-TILM-ERY-LIS-LIN-T/S-TIA	5	2.9
9	PEN-OXA-TLS-TILM-ERY-TET-COL-LIS-TIA	2	1.1
	PEN-OXA-TLS-TILM-ERY-TET-LIS -T/S-TIA	4	2.3
	PEN-OXA-TLS-TILM-ERY-LIN-LIN-T/S-TIA	9	5.1
	PEN-AMX-OXA-TLS-TILM-ERY-LIS-T/S-TIA	11	6.3
8	PEN-AMX-OXA-TLS-ERY-TET-LIS-TIA	2	1.1
	PEN-OXA-TLS-ERY-LIS-LIN-T/S-TIA	2	1.1
	PEN-AMX-OXA-TLS-TILM-ERY-T/S-TIA	3	1.7
	PEN-AMX-OXA-TLS-TILM-ERY-LIS-TIA	4	2.3
	PEN-OXA-TLS-TILM-ERY-LIN-T/S-TIA	5	2.9
	PEN-OXA-TLS-TILM-ERY-LIS-LIN-TIA	5	2.9
	PEN-OXA-TLS-TILM-ERY-LIS-T/S-TIA	26	14.9
7	PEN-OXA-TLS-TILM-ERY-T/S-TIA	5	2.9
	PEN-OXA-TLS-ERY-LIS-T/S-TIA	7	4.0
	PEN-OXA-TLS-TILM-ERY-LIS-TIA	16	9.1
6	PEN-TLS-ERY-LIS-T/S-TIA	2	1.1
	PEN-OXA-TLS-ERY-LIS-T/S	3	1.7
	PEN-OXA-TLS-TILM-ERY-T/S	3	1.7
	PEN-OXA-TLS-TILM-ERY-TIA	4	2.3
	PEN-OXA-TLS-ERY-LIS-TIA	7	4.0
5	PEN-OXA-TLS-ERY-LIS	4	2.3
	PEN-OXA-TLS-ERY-TIA	2	1.1

PEN: penicillin; AMX: amoxicillin; OXA: oxacycline; ENRO: enrofloxacin; CET: ceftiofur; CPP: cefpodoxime-proxetil; CTX: cefotaxime; TLS: tylosin; TILM: tilmicosin; ERY: erythromycin; NEO: neomycin; COL: colistin; TET: tetracycline; DOX: doxycycline; TIA: tiamulin; LIN: lincomycin; LIS: lincomycin-spectinomycin; T/S: trimethoprim-sulfamethoxazole.
